# Prescription n-3 Fatty Acids, But Not Eicosapentaenoic Acid Alone, Improve Reference Memory-Related Learning Ability by Increasing Brain-Derived Neurotrophic Factor Levels in SHR.Cg-*Lepr*^*cp*^/NDmcr rats, A Metabolic Syndrome Model

**DOI:** 10.1007/s11064-013-1121-1

**Published:** 2013-08-21

**Authors:** Michio Hashimoto, Takayuki Inoue, Masanori Katakura, Yoko Tanabe, Shahdat Hossain, Satoru Tsuchikura, Osamu Shido

**Affiliations:** 1 Department of Environmental Physiology, Shimane University Faculty of Medicine, Izumo, Shimane 693-8501 Japan; 2 Department of Biochemistry and Molecular Biology, Jahangirnagar University, Savar, Dhaka, Bangladesh; 3 Disease Model Cooperative Research Association, Hamamatsu, Shizuoka 433-8114 Japan

**Keywords:** Metabolic syndrome, Memory, BDNF, Docosahexaenoic acid, Eicosapentaenoic acid

## Abstract

Metabolic syndrome is implicated in the decline of cognitive ability. We investigated whether the prescription n-3 fatty acid administration improves cognitive learning ability in SHR.Cg-*Lepr* ^*cp*^/NDmcr (SHR-cp) rats, a metabolic syndrome model, in comparison with administration of eicosapentaenoic acid (EPA, C20:5, n-3) alone. Administration of TAK-085 [highly purified and concentrated n-3 fatty acid formulation containing EPA ethyl ester and docosahexaenoic acid (DHA, C22:6, n-3) ethyl ester] at 300 mg/kg body weight per day for 13 weeks reduced the number of reference memory-related errors in SHR-cp rats, but EPA alone had no effect, suggesting that long-term TAK-085 administration improves cognitive learning ability in a rat model of metabolic syndrome. However, the working memory-related errors were not affected in either of the rat groups. TAK-085 and EPA administration increased plasma EPA and DHA levels of SHR-cp rats, associating with an increase in EPA and DHA in the cerebral cortex. The TAK-085 administration decreased the lipid peroxide levels and reactive oxygen species in the cerebral cortex and hippocampus of SHR-cp rats, suggesting that TAK-085 increases antioxidative defenses. Its administration also increased the brain-derived neurotrophic factor levels in the cortical and hippocampal tissues of TAK-085-administered rats. The present study suggests that long-term TAK-085 administration is a possible therapeutic strategy for protecting against metabolic syndrome-induced learning decline.

## Introduction

Metabolic syndrome as a whole and several of its components have a negative impact on cognitive function [1, 2] in elderly individuals who are usually vulnerable to age-related neurodegenerative diseases such as Alzheimer’s disease (AD) [3] and vascular dementia [4]. Epidemiological studies support that modifiable vascular and lifestyle-related factors are associated with the development of dementia and predementia syndromes in late life, and these studies identified multiple potentially preventable risk factors [5]. In particular, vascular-related factors such as high blood pressure and hypertension, total cholesterol and other lipid parameters, diabetes and insulin resistance, body mass index, obesity, and metabolic syndrome have been associated with dementia and cognitive decline [6, 7]. Thus, people with metabolic syndrome are more likely to experience decline in memory than those without the syndrome. Because metabolic syndrome and its components are potentially modifiable, it would be possible for treatment to prevent cognitive decline, and thus prevent dementia.

Docosahexaenoic acid (DHA, C22:6, n-3) and eicosapentaenoic acid (EPA, C20:5, n-3) are the primary n-3 polyunsaturated fatty acids (PUFAs) in fish oil. Epidemiological studies revealed that fish oil intake is associated with reduced risk of neurological and psychiatric disorders. In addition, van Gelder et al. [8] examined cognitive decline over a 5-year period and reported that increase in fish consumption and DHA + EPA intake are both associated with reduction in cognitive decline. Moreover, fish consumption and n-3 PUFA intake are associated with reduced risk of cognitive decline and dementia [9]. It has been very recently reported that daily DHA and EPA supplementation has beneficial effects against age-related cognitive decline in otherwise health elderly Japanese individuals with very mild dementia [10]. These findings suggest that increased consumption of n-3 PUFAs is associated with reduced risk of age-related cognitive decline, dementia, and Alzheimer’s disease.

DHA is one of the primary essential fatty acids in the human brain, and it is present at very high concentrations in neural synaptosomal plasma membranes and synaptic vesicles. DHA accrues in the developing brain during the brain growth spurt [11], and DHA deficiency impairs memory and learning and promotes age-related neurodegenerative diseases [12]. Although DHA is essential for various neural functions, the DHA biosynthetic pathway does not produce the sufficient amount of DHA required for normal brain functioning. Because vertebrates do not have adequate metabolic capacity to insert double bonds in the appropriate positions, they are dependent on the diet to supply this fatty acid. These results have raised the possibility whether administration of the DHA precursor, i.e., EPA, could purposefully be used for the expected neurobehavioral outcome of DHA. The dietary supplementation of DHA ameliorates the learning-related spatial memory of rats [13–16]. Moreover, EPA administration increased neuronal and glial EPA content and glial DHA content, suggesting that EPA may protect against neurodegeneration by modulating synaptic plasticity [17]. In addition, dietary EPA administration increases the DHA levels and the DHA/arachidonic acid (AA) ratio in the plasma and brain tissues of normal or amyloid β (Aβ)-infused AD rats in association with decrease in oxidative stress [18]. From these results, it is demonstrated that EPA and/or DHA could be used to prevent memory deficits.

In this study, using SHR.Cg-*Lepr* ^*cp*^/NDmcr (SHR-cp) rats, a metabolic syndrome model, we investigated whether the prescription administration of n-3 fatty acids (TAK-085: highly purified and concentrated EPA + DHA ethyl esters) or EPA alone improves cognitive learning ability in rats with metabolic abnormalities. Spontaneously hypertensive rats (SHR) exhibit impaired performance of both spatial and nonspatial learning and memory-related task [19–21]. SHR-cp rats derived from SHR spontaneously develop obesity, hypertension, hyperlipidemia, hyperglycemia, and hyperinsulinemia, i.e., metabolic syndrome [22, 23]. Metabolic syndrome might also impose a serious metabolic threat to brain activities such as the process of learning that encodes for memory. Thus, this rat model appears well suited for assessing the changes induced by broad metabolic abnormalities and the development of memory loss. We finally evaluated whether TAK-085 affects memory-related spatial task and the underlying mechanisms.

## Materials and Methods

Five-week-old male SHR-cp rats were supplied by the Disease Model Cooperative Research Association (Kyoto, Japan). The rats were housed in an air-conditioned animal room with a 12:12-h dark:light cycle under controlled temperature (23 ± 2 °C) and relative humidity (50 ± 10 %). After acclimatization, they were randomly divided into three groups–the control rats (n = 11), TAK-085-treated rats (n = 11), and EPA-treated rats (n = 11). The rats were provided with a high cholesterol-containing diet pellet (a standard F1 pellet containing no fish products and including 1 % cholesterol and 0.3 % cholic acid; Funabashi Farm, Funabashi, Japan; Table 1) and water ad libitum. All animal experiments were performed in accordance with the procedures outlined in the Guidelines for Animal Experimentation of Shimane University compiled from the Guidelines for Animal Experimentation of the Japanese Association for Laboratory Animal Science. The TAK-085-treated rats were orally administered TAK-085 (300 mg/kg body weight per day: Pronova BioPharma ASA, Oslo, Norway) containing 498 mg/g EPA, 403 mg/g DHA, and 4.8 mg/g α-tocopherol suspended in 5 % gum Arabic solution for 13 weeks; EPA rats were administered EPA-E (300 mg/kg body weight per day; Nisshin Pharma Inc., Tokyo, Japan) containing 980 mg/g EPA and 1.9 mg/g α-tocopherol suspended in 5 % gum Arabic solution for 13 weeks; and control rats were administered 5 % gum Arabic solution containing 4.8 mg/g α-tocopherol for 13 weeks. TAK-085 and EPA were gently emulsified in a 5 % gum Arabic solution in an ultrasonic cell homogenizer (Taitec VP-5; Taitec, Tokyo, Japan) immediately before administration. Administration was maintained until all experiments had been completed.

**Table 1:** Components of a high-cholesterol diet and TAK-085 profiles

HC diet		Profiles of TAK-085	
Composition of the diet (%, w/w)		Eicosapentaenoic acid _C20:5(n-3)_ (EE) (mg/g)	462
Water	8.0	Docosahexaenoic acid _C22:6(n-3)_ (EE) (mg/g)	367
Crude protein	21.5	EPA and DHA (mg/g)	829
Fat	4.4	Docosapentaenoic acid _C22:5(n-3)_ (%, w/w)	3.3
Fiber	2.6	Total n-3 (EE) (%, w/w)	90
Mineral	4.9	Arachidonic acid _C20:4(n-6)_ (EE) (%, w/w)	2.4
Carbohydrate	58.6	Docosapentaenoic acid _C22:5(n-6)_ (%, w/w)	1.0
Cholesterol	1.0	α-Tocopherol (mg/g)	3.9
Cholic acid	0.3		
Fatty acid composition (g/kg)			
Myristic acid _C14:0_	0.034		
Palmitic acid _C16:0_	5.83		
Palmitoleic acid _C16:1(n-7)_	ND		
Stearic acid _C18:0_	2.24		
Oleic acid _C18:1(n-9)_	8.57		
Linoleic acid _C18:2(n-6)_	21.5		
Linolenic acid _C18:3(n-3)_	2.21		
Arachidonic acid _C20:4(n-6)_	ND		
Eicosapentaenoic acid _C20:5(n-3)_	ND		
Docosapentaenoic acid _C22:5(n-3)_	ND		
Docosahexaenoic acid _C22:6(n-3)_	ND		
Lignoceric acid _C24:0_	0.055		

*DHA* docosahexaenoic acid, *EE* ethyl ester, *EPA* eicosapentaenoic acid, *ND* not detected

The high-cholesterol diet, which is the standard F1 diet containing no fish products, contained 1 % cholesterol and 0.3 % cholic acid, and it was purchased from Funabashi Farm, Chiba, Japan

### Eight-Arm Radial Maze Task

Seven weeks after the start of TAK-085/EPA administration, the rats’ learning-related behavior was assessed by their completion of a task in an eight-arm radial maze as previously described [13, 15]. The rats were placed on a food deprivation regimen that reduced their body weight to 70–75 % of the free-feeding weight and were handled for 5 min daily for 5 consecutive days. The radial maze was placed in a closed room with a number of visual cues: fluorescent ceiling lights, curtained door, a chair for the observer and some boxes. The experimenter maintained a constant position beside the maze and observed the behavior of the rats. Then for 5 days, the rats were familiarized with the apparatus in which 45 mg reward pellets (made with F1) were scattered throughout the maze. Each rat was tested by two daily trials for 6 days/week for a total of 5 weeks. The trial consisted of baiting only four of the arms (consistently the same arm for any one animal) with reward pellets and placing the rat in the center of the platform facing a randomly selected arm. Two parameters of memory function were examined—(1) reference memory error (RME), determined by the number of entries into the unbaited arms, and (2) working memory error (WME), estimated by the number of repeated entries into arms that had already been visited during the trial. Memory-related behavior was calculated on the basis of the performance in the maze arms.

### Sample preparation

After completing the behavioral studies, the rats were anesthetized with sodium pentobarbital (65 mg/kg BW, intraperitoneally), blood was collected, and the cerebral cortex and hippocampus were separated as described previously [15]. The tissues were stored at −80 °C by flash-freezing in liquid N_2_ until use or immediately homogenized in ice-cold 0.32 mol/L sucrose buffer (pH 7.4) containing 2 mmol/L EDTA, 0.5 mg/L leupeptin, 0.5 mg/L pepstatin, 0.5 mg/L aprotinin, and 0.2 mmol/L phenylmethylsulfonyl fluoride using a Polytron homogenizer (PCU 2–110; Kinematica). The homogenates were immediately subjected to additional assays or stored at −80 °C after a liquid N_2_ flash and bath until use.

### Measurement of Brain-Derived Neurotrophic Factor (BDNF)

The whole homogenate was centrifuged at 13,000 × *g* for 30 min, and the resulting supernatant was used for BDNF assays. BDNF was quantified using an enzyme-linked immunosorbent assay kit (BDNF Emax ImmunoAssay System kit, Promega Inc., Madison, WI) according to the manufacturer’s protocol. The BDNF levels were calculated in pg/mg of cytosolic protein and reported as % of control.

### Measurement of Oxidative Stress and Fatty Acid Profiles

Reactive oxygen species (ROS) levels were determined as described previously by Hashimoto et al. [14]. In brief, 50 μL of freshly prepared tissue homogenate was mixed with 4.85 mL of 100 mmol/L potassium phosphate buffer (pH 7.4) and incubated with 2′,7′-dichlorofluorescin diacetate in methanol at a final concentration of 5 μmol/L for 15 min at 37 °C. The dye-loaded samples were centrifuged at 12,500×*g* for 10 min at 4 °C. The pellet was mixed on a vortex at 0 °C in 5 mL of 100 mmol/L phosphate buffer (pH 7.4) and incubated again for 60 min at 37 °C. Fluorescence was measured with a Hitachi 850 spectrofluorometer (Tokyo, Japan) at wavelengths of 488 nm for excitation and 525 nm for emission. The cuvette holder was maintained at 37 °C. ROS was quantified using a dichlorofluorescein standard curve in methanol.

Lipid peroxide (LPO) concentrations were assessed by the thiobarbituric acid reactive substance (TBARS) assay, as described previously [14]. The TBARS levels were measured in nanomoles of malondialdehyde/mg protein. Malondialdehyde levels were calculated relative to a standard preparation of 1,1,3,3-tetraethoxypropane.

The fatty acid compositions of plasma and brain tissues were determined using a modification of the one-step reaction of Lepage and Roy [24] by gas chromatograpy as described previously [14]. Protein concentrations were estimated by the method of Lowry et al. [25].

### Statistical analysis

Results are expressed as mean ± SEM. Behavioral data were analyzed by a two-factor (group and block) randomized block factorial ANOVA, and all other parameters were analyzed for intergroup differences by one-way ANOVA. ANOVA was followed by Fisher’s PLSD for post hoc comparisons. Correlations were determined by simple regression analysis. The statistical programs used were GB-STAT™ 6.5.4 (Dynamic Microsystems) and Stat-View^®^ 4.01 (MindVision Software, Abacus Concepts). Differences with *P* < 0.05 were considered significant.

## Results

### Body Weight

Final body weights did not differ among the three groups (control group: 489 ± 9 g; TAK-085: 496 ± 5 g; EPA: 500 ± 4 g).

### Effect of TAK-085 and EPA Administration on Radial-Maze Learning Ability

**Fig. 1:**
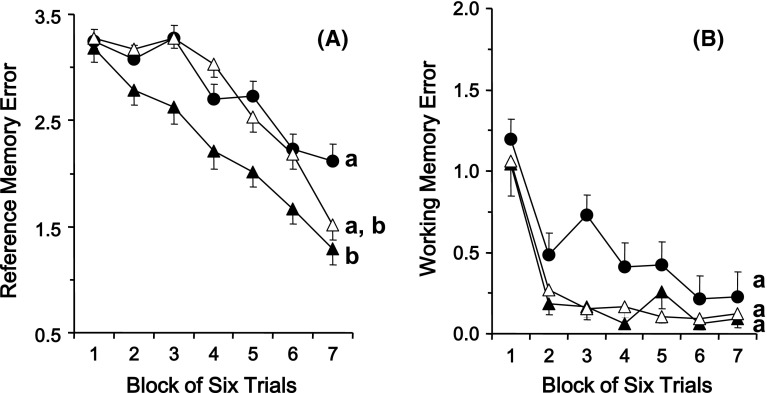
Effect of long-term TAK-085 and EPA administration on the reference (**a**) and working (**b**) memory-related learning ability of the SHR-cp rats in the radial maze task. (*filled circle*) Control rats (n = 11); (*filled triangle*) TAK-085-treated rats (n = 11); (*open triangle*) EPA-treated rats (n = 11). Each *value* represents the number of RMEs and WMEs as the mean ± SEM in each block of six trials. The main effects of the blocks of trial and groups are indicated in the “Results” section. The significance of the differences among the three groups was determined by randomized two-factor (block and group) ANOVA followed by the Bonferroni post hoc test. Groups without a *common alphabet* for the main effects of groups are significantly different at *P* < 0.05. Details of the subtest analysis between the two groups of the main effects of blocks of trials, groups, and block × group interaction are indicated in Table 2

The effects of long-term administration of TAK-085 and EPA alone on reference and working memory-related learning abilities are presented as the mean number of RMEs and WMEs for each group with data averaged over blocks of six trials in the Fig. 1a, b, respectively. Randomized two-factor (block and group) ANOVA revealed a significant main effect of both groups (*F*_2,20_ = 5.97, *P* = 0.009) and blocks of trials (*F*_6,60_ = 35.52, *P* < 0.001) with a significant group × block interaction (*F*_12,120_ = 1.85, *P* = 0.047) on the number of RMEs (Fig. 1a). Regarding the WMEs (Fig. 1b), randomized two-factor (block and group) ANOVA revealed a significant main effect of both groups (*F*_2,20_ = 4.07, *P* = 0.033) and blocks of trials (*F*_6,60_ = 29.20, *P* < 0.001) without a significant group × block interaction (*F*_12,120_ = 0.709, *P* = 0.740).

Subtest analyses (Table 2) of the RMEs and WMEs revealed the effect of TAK-085 or EPA on SHR-cp rats. Subtest analysis revealed a significant effect of TAK-085 on control rats [RMEs: groups (*P* = 0.026) and blocks of trials (*P* < 0.001) with a tendency of significant group × block interaction (*P* = 0.052); WMEs: groups (*P* = 0.047) and blocks of trials (*P* < 0.001) but without a significant group × block interaction (*P* = 0.547)]. These analyses demonstrated that the number of RMEs, but not WMEs, tended to be significantly lower in the TAK-085-administered rats than in the control rats (Fig. 1). Whereas, subtest analysis revealed no significant effect of EPA on control rats [RMEs: groups (*P* = 0.726) and blocks of trials (*P* < 0.001) without a significant group × block interaction (*P* = 0.128); WMEs: groups (*P* = 0.056) and blocks of trials (*P* < 0.001) but without a significant group × block interaction (*P* = 0.518)]. These analyses demonstrated that there were no statistically significant differences in the number of RMEs and WMEs between the EPA-treated rats and the control rats (Fig. 1). Subtest analysis also revealed no significant differences between the TAK-085- and EPA-treated rats regarding RMEs and WMEs [RMEs: groups (*P* = 0.012) and blocks of trials (*P* < 0.001) without a significant group × block interaction (*P* = 0.140), WMEs: groups (*P* = 0.836) and blocks of trials (*P* < 0.001) without a significant group × block interaction (*P* = 0.937)]. These analyses demonstrated that there was no significant difference in the number of RMEs and WMEs between the TAK-085- and EPA-treated rats (Fig. 1). These results finally suggest that long-term administration of TAK-085, but not EPA alone, improved reference memory-related learning ability but not working memory-related learning ability in the SHR-cp rats.

**Table 2:** Results of the two-factor ANOVA and PLSD test conducted on RME and WME data obtained from the control (n = 11), TAK-085-treated (n = 11), and EPA-treated (n = 11) groups

	Group	Block	Group × Block
*Reference memory error*			
Control versus TAK-085	0.026 [F(1, 10) = 6.85]	<0.001 [F(6,60) = 17.62]	0.052 [F(6,60) = 2.23]
Control versus EPA	0.726 [F(1, 10) = 0.13]	<0.001 [F(6,60) = 28.77]	0.128 [F(6,60) = 1.74]
TAK-085 versus EPA	0.012 [F(1,10) = 9.31]	<0.001 [F(6,60) = 41.01]	0.140 [F(6,60) = 1.69]
*Working memory error*			
Control versus TAK-085	0.047 [F(1,10) = 5.14]	<0.001 [F(6,60) = 16.05]	0.549 [F(6,60) = 0.833]
Control versus EPA	0.056 [F(1,10) = 4.68]	<0.001 [F(6,60) = 18.54]	0.518 [F(6,60) = 0.876]
TAK-085 versus EPA	0.836 [F(1,10) = 0.045]	<0.001 [F(6,60) = 22.33]	0.937 [F(6,60) = 0.937]

These data are also presented in Fig. 1

### Effect on BDNF

**Fig. 2:**
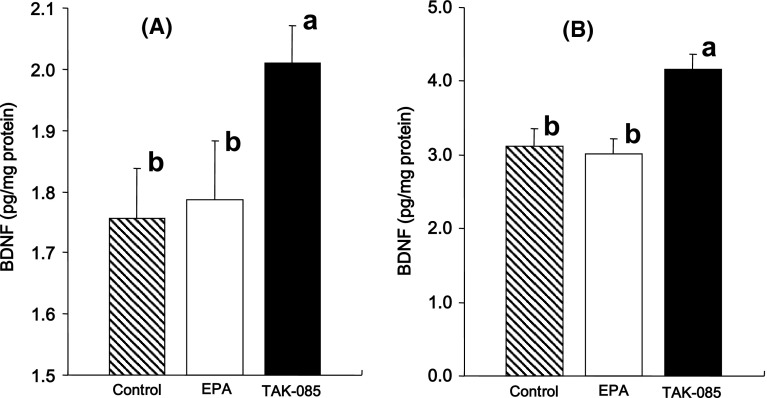
Effect of long-term TAK-085 and EPA administration on the levels of brain-derived neurotrophic factor (BDNF) levels in the cerebral cortex (**a**) and hippocampus (**b**) of the control, EPA-treated, and TAK-085-treated rats. Data are presented as the mean ± SEM. (*shaded square*), Control rats (n = 11); (*open squre*), EPA-treated rats (n = 11); (*filled square*), TAK-085-treated rats (n = 11). *Bars* without a *common alphabet* are significantly different at *P* < 0.05. Data were analyzed by one-way ANOVA followed by Fisher’s PLSD post hoc for multiple comparisons

The BDNF levels in the TAK-085 rats were increased by 15 % (*F*_1,20_ = 7.22, *P* = 0.014) in the cerebral cortex (Fig. 2a) and by 34 % (*F*_1,20_ = 12.05, *P* = 0.0027) in the hippocampus (Fig. 2b) compared to those in control rats. There were no statistical significant differences in the cerebrocortical and hippocampal BDNF levels between the control and EPA-treated rats and between the EPA- and TAK-085-treated rats (Fig. 2).

### Oxidative Stress in the Plasma and Brain

**Fig. 3:**
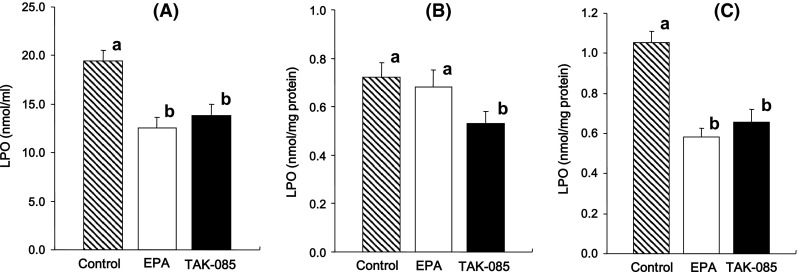
Effect of long-term TAK-085 and EPA administration on the lipid peroxide (LPO) levels in the plasma (**a**), cerebral cortex (**b**) and hippocampus (**c**) of the SHR-cp rats. Data are presented as the mean ± SEM. (*shaded square*) Control rats (n = 11); (*open squre*) EPA-treated rats (n = 11); (*filled square*) TAK-085-treated rats (n = 11). *Bars* without a *common alphabet* are significantly different at *P* < 0.05. Data were analyzed by one-way ANOVA followed by Fisher’s PLSD post hoc for multiple comparisons

Plasma LPO levels were significantly lower in the EPA- and TAK-085-treated rats than in the control rats, but no statistical significance was found between the EPA- and TAK-085-treated rats (*F*_2,30_ = 11.62, *P* = 0.0002) (Fig. 3a). The LPO levels in the cortex were significantly lower in the TAK-085-treated rats (*F*_1,20_ = 6.32, *P* = 0.02) than in the control rats; however, there was no statistical significant difference between the EPA-treated and control rats (Fig. 3b). The LPO levels in the hippocampus were significantly lower in the EPA- and TAK-085-treated rats than in the control rats (*F*_2,30_ = 22.49, *P* < 0.0001), but there was no significant difference between the EPA- and TAK-085-treated rats (Fig. 3c).

**Fig. 4:**
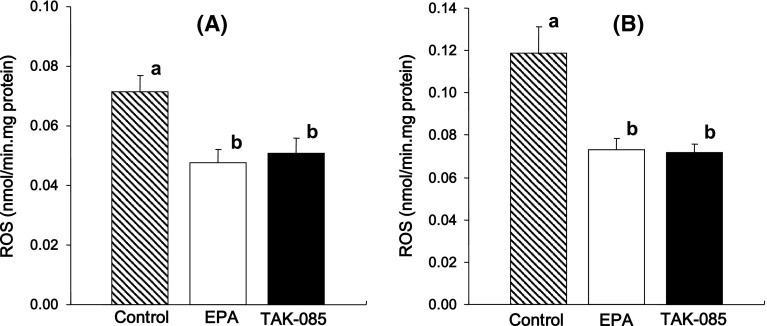
Effect of oral TAK-085 and EPA administration on the reactive oxygen species (ROS) levels in the cerebral cortex (**a**) and hippocampus (**b**) of the control, EPA-treated, and TAK-085-treated rats. Data are presented as the mean ± SEM. (*shaded square*), Control rats (n = 11); (*open squre*), EPA-treated rats (n = 11); (*filled square*), TAK-085-treated rats (n = 11). *Bars* without a *common alphabet* are significantly different at *P* < 0.05. Data were analyzed by one-way ANOVA followed by Fisher’s PLSD post hoc for multiple comparisons

The ROS levels were 31 and 32 % lower in the cerebral cortices of EPA- and TAK-085-treated rats, respectively (*F*_2,30_ = 6.4, *P* = 0.0048) (Fig. 4a), and 38 and 39 % lower, respectively (*F*_2,30_ = 11.69, *P* = 0.0001) in the hippocampus (Fig. 4b) than those of the control rats. There were no statistically significant differences in the ROS levels in the cerebral cortex and hippocampus between the EPA- and TAK-085-treated rats.

### Plasma and Brain Fatty Acid Profiles

The plasma fatty acid profiles of the rats are shown in Table 3. The plasma levels of EPA, DHA and docosapentaenoic acid [DPA, C22:5(n-3)] were significantly higher in both the TAK-085- and EPA-treated rats than in the control rats, but those of AA were significantly lower in the TAK-085- and EPA-treated rats than in the control rats. The plasma EPA and DPA levels were significantly higher in the EPA-treated rats than in the TAK-085-treated rats, and the DHA levels were higher in the TAK-085-treated rats than in the EPA-treated rats. The plasma DHA levels were significantly higher in the TAK-085-treated rats than in both the EPA-treated and control rats; similarly, the DHA levels (*P* = 0.0835) tended to be higher in the EPA-treated rats than in the control rats. The plasma levels of stearic acid were significantly higher in the TAK-085- and EPA-treated rats than in the control rats, but its levels did not differ between the TAK-085- and EPA-treated rats. TAK-085 and EPA administration significantly increased the plasma DHA/AA molar ratio; however, their administration did not affect the plasma levels of palmitic acid, oleic acid, linoleic acid, or linolenic acid.

**Table 3:** Plasma fatty acid profiles

	Control (n = 11)	TAK-085 (n = 11)	EPA (n = 11)
Palmitic acid _C16:0_	1,036 ± 61	1,047 ± 87	877 ± 64
Stearic acid _C18:0_	359 ± 13^a^	299 ± 17^b^	257 ± 14^b^
Oleic acid _C18:1(n-9)_	1,232 ± 76	1,181 ± 114	947 ± 86
Linoleic acid _C18:2(n-6)_	596 ± 46	717 ± 56	601 ± 48.6
Linolenic acid _C18:3(n-3)_	13.8 ± 1.6	18.8 ± 2.0	15.9 ± 1.4
Arachidonic acid _C20:4(n-6)_	1,146 ± 50^a^	644 ± 36^b^	528 ± 34^b^
Eicosapentaenoic acid _C20:5(n-3)_	22.8 ± 1.7^c^	118 ± 5.3^b^	158 ± 11.4^a^
Docosapentaenoic acid _C22:5(n-3)_	44.7 ± 3.9^c^	70.8 ± 6.2^b^	102.2 ± 9.2^a^
Docosahexaenoic acid _C22:6(n-3)_	49.0 ± 3.2^c^	237 ± 20.6^a^	81.0 ± 6.7^b^
C22:6(n-3)/C20:4(n-6)	0.04 ± 0.00^c^	0.35 ± 0.03^a^	0.14 ± 0.01^b^

The fatty acid values are expressed as μg/mL; values are mean ± SEM; Means in a row with superscripts without a common alphabet differ at *P* < 0.05

The major fatty acid profiles in the rat cerebral cortex and hippocampus are shown in Table 4. The EPA and DHA levels in the cerebral cortex were significantly higher in both the TAK-085- and EPA-treated rats than in the control rats, but the AA levels did not differ, causing a significant increase in the DHA/AA molar ratio in the cerebral cortex. EPA administration significantly increased the hippocampal EPA levels compared with those in the control rats, whereas the hippocampal EPA levels (*P* = 0.0792) tended to be higher in TAK-085-treated rats than in the control rats. TAK-085 and EPA administration did not affect the DHA and AA levels in the hippocampus.

**Table 4:** Major fatty acid levels of the cerebral cortex and hippocampus

	Control (n = 11)	TAK-085 (n = 11)	EPA (n = 11)
*Cerebral cortex*			
Arachidonic acid _C20:4(n-6)_	28.45 ± 1.98	27.76 ± 2.74	30.28 ± 4.54
Eicosapentaenoic acid _C20:5(n-3)_	0.14 ± 0.01^b^	0.30 ± 0.05^a^	0.34 ± 0.06^a^
Docosahexaenoic acid _C22:6(n-3)_	43.24 ± 2.45^b^	54.5 ± 5.96^a^	53.27 ± 7.11^a^
C22:6(n-3)/C20:4(n-6)	1.42 ± 0.04^c^	1.81 ± 0.05^a^	1.66 ± 0.04^b^
Hippocampus			
Arachidonic acid _C20:4(n-6)_	39.69 ± 3.63	35.07 ± 4.73	41.04 ± 5.82
Eicosapentaenoic acid _C20:5(n-3)_	0.27 ± 0.03^b^	0.37 ± 0.05^b^	0.50 ± 0.05^a^
Docosahexaenoic acid _C22:6(n-3)_	46.12 ± 3.58	49.0 ± 5.84	52.84 ± 6.48
C22:6(n-3)/C20:4(n-6)	1.10 ± 0.07^b^	1.32 ± 0.07^a^	1.19 ± 0.04^a,b^

The fatty acid values are expressed as μg/mg protein; values are mean ± SEM; Means in a row with superscripts without a common alphabet differ at *P* < 0.05

### Correlation Between Cognitive Function, Corticohippocampal BDNF Levels and the DHA/AA Molar Ratio

**Fig. 5:**
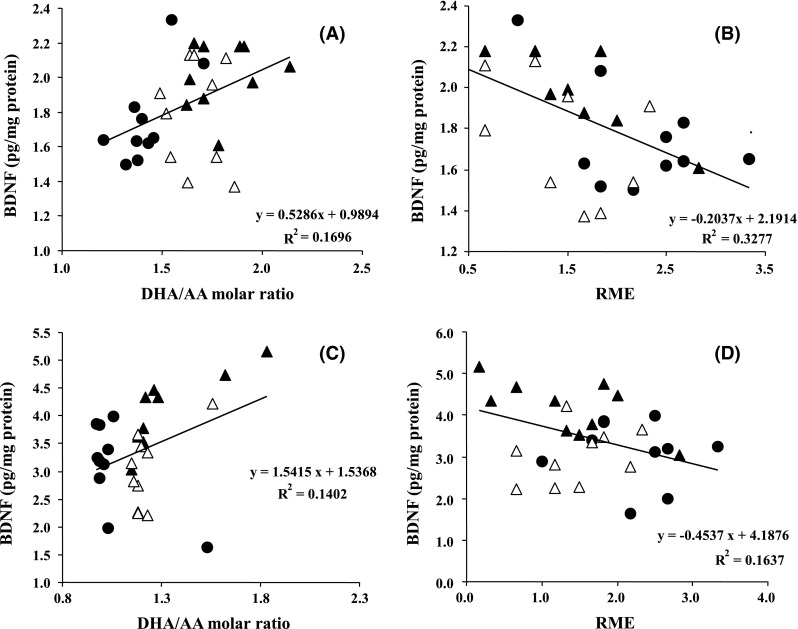
Correlation between the learning ability and the BDNF levels and the DHA/AA molar ratio in cortical (Fig. 5a, b) and hippocampal (Fig. 5c, d) tissues. The number of RMEs in block 7 shown in Fig. 1 was used as an indicator of learning ability. Data were analyzed by simple regression analysis. (*filled circle*), Control rats (n = 11); (*filled triangle*), TAK-085-treated rats (n = 11); (*open square*), EPA-treated rats (n = 11)

To define the relationship of learning and memory with the BDNF levels, we assessed the correlation between performance in the radial arm maze and the BDNF levels and the molar DHA/AA ratios in corticohippocampal tissues. Regression analyses revealed significant positive correlations between the BDNF levels and the DHA/AA molar ratios in both the cortex (r^2^ = 0.170, *P* = 0.024) (Fig. 5a) and hippocampus (r^2^ = 0.140, *P* = 0.045) (Fig. 5c) and negative correlations between the number of RMEs in the final block of the radial maze task and the BDNF levels in both the cerebral cortex (r^2^ = 0.328, *P* < 0.001) (Fig. 5b) and hippocampus (r^2^ = 0.164, *P* = 0.027) (Fig. 5d). In addition, when all the corticohippocampal data were analyzed, the DHA/AA molar ratio was negatively correlated with the numbers of RMEs in the final block of the radial maze task (r^2^ = 0.148, *P* = 0.0017), the cortico-hippocampal LPO levels (r^2^ = 0.155, *P* = 0.0013) and the corticohippocampal ROS levels (r^2^ = 0.232, *P* < 0.0001).

## Discussion

This study examined the effect of n-3 PUFA administration, including differences in the quantity of EPA and DHA, on the learning processes and memory in SHR-cp rats and the plausible underlying mechanism of actions with an emphasis on EPA and DHA partitioning in the plasma as well as the cerebral cortex and hippocampus, the most important brain regions responsible for memory formation. There were significant differences in sensitivity and n-3 PUFA-induced changes in the learning-related memory ability of the SHR-cp rats.

In this study, TAK-085 containing 50 % EPA and 40 % DHA had a more pronounced influence on reference memory-related learning ability than EPA alone. EPA comprises only a small amount of total PUFAs in the brain compared to the DHA levels (Table 4). The EPA levels were increased in the cortex and hippocampus of EPA- and TAK-085-treated SHR-cp rats, although the total levels (i.e. even after increase) remained very low compared to the DHA levels. This increase could not be attributed to a metabolic conversion from α-linolenic acid because the levels of this fatty acid were not altered in the cortex or hippocampus of EPA- or TAK-085-treated rats (data not shown). Rather, this increase may be attributable to retroconversion from DHA via DPA. Thus, the question is whether the magnitude of increase in the EPA levels (0.1–0.3/0.3 in the cortex or 0.3–0.5/0.4 in the hippocampus) can be explained by EPA-induced alterations in the molecular composition/systems of corticohippocampal neurons and the resultant spatial cognition. Long-term administration of EPA ameliorated the spatial learning ability in normal Wistar rats and significantly increased corticohippocampal DHA levels [18]. This may relate to the fact that ^14^C-labeled EPA levels in the rat brain decreases time dependently beginning 1 h after its oral administration, whereas those of [^14^C]DHA, a metabolite of EPA, increase time dependently [26], indicating that neuronally available EPA is continuously being subjected to conversion into DHA. Despite the increases in the levels of EPA in the plasma and/or brains of EPA-treated rats, unfortunately, SHR-cp rats failed to demonstrate improvements of spatial memory (Fig. 1a). This discrepancy may be resulted from the fact that we used metabolic syndrome model rats instead of normal rats.

Dietary EPA and DHA were recently claimed to affect biological activities differently. A meta-analysis of clinical trials revealed that EPA rather than DHA ameliorates depression, presumably by the peripheral anti-inflammatory effect of EPA [27]. EPA rather than DHA appears to be more effective in ameliorating attention/deficit hyperactivity disorder [28]. Age-related decreases in neuronal inflammation are overcome by supplementation with EPA [29]. Very recently, it was reported that DHA, but not EPA, reduces early inflammatory responses following spinal cord injury in rats [30]. DHA-induced alterations in bilayer acylchain properties and functions such as phase behavior, elastic compressibility, ion permeability, fusion, flip-flop, and resident protein functions and enzyme activities underlie its pleiotropic salutary effects [31]. Consistent with the aforementioned reports, DHA-induced changes in neuronal membrane properties are correlated with memory-related learning ability [32]. Moreover, long-term DHA administration positively affects vascular biology [33, 34]. EPA and DHA have different metabolic and physiological effects in humans. From these reports, it must be distinguished whether combined treatment with EPA and DHA or individual administration of each fatty acid provides greater benefits [35]. TAK-085-treated SHR-cp rats displayed improved performances relative to that of control SHR-cp rats at most of the blocks (Fig. 1a). In contrast, no differences were found between the control and EPA-treated SHR-cp rats (Fig. 1b), whereas EPA significantly ameliorated the spatial memory of normal and Aβ_1–40_-infused Alzheimer’s disease model rats [18]. Therefore, the sensitivity of rats to EPA administration may be related to the discrepancies of the outcome of EPA administration.

Dietary n-3 PUFA deprivation, particularly that of DHA, decreases the levels of BDNF, which increases neuroplasticity and cell survival [36, 37], in the frontal cortex of rats [38]. BDNF is implicated in the pathophysiology of several neuropsychiatric disorders [39] and reductions in the BDNF levels in the hippocampus impair learning and memory in animals. These findings led us to investigate the influences of TAK-085 and EPA on the BDNF levels in the corticohippocampal regions of the SHR-cp rats. In this study, the BDNF levels were significantly increased in both the cerebral cortex and hippocampus of TAK-085-treated rats (Fig. 2). This appears consistent with the findings of increased levels of BDNF in the DHA-treated rats [38]. It can be speculated that the ameliorative effect of TAK-085 on cognitive learning ability is related to the increased BDNF levels in the brains of the TAK-085-treated rats. More importantly, the DHA/AA molar ratio, which is positively correlated with the spatial memory of rats [13–15], was increased significantly in both the cerebral cortex and hippocampus of the TAK-085-treated rats (Table 4). Thus, consistent with our previous reports, it is again postulated that the DHA/AA molar ratio is positively correlated with both the BDNF levels and the learning ability (the reciprocal of RME is memory) in the SHR-cp rats (Fig. 5). BDNF acts as a memory molecule in that it increases long-term potentiation (LTP) [40], neurochemical substrate and foundation of synaptic plasticity, and memory formation [41]. Administration of DHA to the n-3 PUFA-deprived rats enhances the learning ability [13–15, 42–44], and prevents cognitive declines [14, 15, 32], probably by reversing synaptic impairments such as those in LTP [45, 46], and stimulating in vitro and in vivo neurogenesis [47, 48], and c-fos activation [42].

DHA reduces oxidative stress [14, 15, 49, 50]. ROS-induced traumatic brain injury is associated with reduction in the BDNF levels [51]. Hou et al. [52] reported that oral administration of hydrogen-rich water improves BDNF attenuation-related cognitive deficits. Dietary DHA increases the BDNF levels with concomitant improvement in traumatic brain injury-induced water maze memory deterioration and oxidative stress) [53]. These reports all corroborate our speculation that the TAK-085-induced increases in the BDNF levels might be achieved, at least partially, through the inhibitory effect of DHA of TAK-085 on oxidative stress. TAK-085 supplementation reduced the elevated LPO and ROS levels in the SHR-cp rats (Figs. 2, 3). It is thus conceivable that the potential antioxidant action of DHA in the TAK-085-treated rats occurs through mechanisms that maintain synaptic plasticity and increase memory ability. In other words, TAK-085 counteracted the elevated LPO/ROS levels with subsequent effects on BDNF-mediated effects on synaptic plasticity and cognition.

Moreover, long-term EPA administration has a neuroprotective effect on the modulation of rat hippocampal synaptic plasticity by both its capacity to increase brain DHA levels and its direct effects on neurons and glial cells [17]. Thus, it is suggested that TAK-085 is more effective than DHA or EPA alone for preventing metabolic syndrome- and/or age-related cognitive decline. Finally, n-3 PUFA-induced improvements in memory and learning are believed to be underpinned by various factors, including antioxidative effects, stimulation of hippocampal neurogenesis, and modulation of neuronal signaling pathways. The present experiments may provide such novel evidence that the beneficial effects of DHA on cognitive impairment in rats with metabolic syndrome is associated with the restoration of molecular systems, including BDNF, which regulates synaptic plasticity to enhance memory. Irrespective of the mechanism(s), this study demonstrated that TAK-085 containing EPA and DHA displayed more beneficial effects on the spatial learning ability of rats with metabolic syndrome than EPA alone.

In summary, TAK-085 significantly improved reference memory-related learning ability in SHR-cp rats. The beneficial effects of TAK-085 supplementation, particularly in the brains of SHR-cp rats, might be attributable to DHA, which was transformed from its precursor EPA and obtained from dietary sources. This possibility is supported by the fact that EPA is absent from the brain or present in small amounts. To more greatly affect and/or strongly correlate with the functions of neurons and related neurobehavioral aspects of rats, EPA must be physically present in the brain: we believe that at least partially, all the effects of EPA administration on brain function must be because of its metabolite DHA. Further studies are essential, particularly to evaluate the effects of EPA versus DHA by studying all of their possible active metabolites.

## Abbreviations

Aβ: Amyloid β
AA: Arachidonic acid
BDNF: Brain-derived neurotrophic factor
DHA: Docosahexaenoic acid
DPA: Docosapentaenoic acid
EPA: Eicosapentaenoic acid
LPO: Lipid peroxide
LTP: Long-term potentiation
PUFA: Polyunsaturated fatty acid
RME: Reference memory error
ROS: Reactive oxygen species
SHR-cp: SHR.Cg-*Lepr* ^*cp*^/NDmcr
TBARS: Thiobarbituric acid reactive substance
WME: Working memory error

## References

[1] Yaffe K, Haan M, Blackwell T, Cherkasova E, Whitmer RA, West N (2007). Metabolic syndrome and cognitive decline in elderly Latinos: findings from the Sacramento Are Latino Study of Aging study. J Am Geriatr Soc.

[2] Komulainen P, Lakka TA, Kivipelto M, Hassinen M, Helkala EL, Haapala I, Nissinen A, Rauramaa R (2007). Metabolic syndrome and cognitive function: a population-based follow- up study in elderly women. Dement Geriatr Cogn Disord.

[3] Vanhanen M, Koivisto K, Moilanen L, Helkala EL, Hänninen T, Soininen H, Kervinen K, Kesäniemi YA, Laakso M, Kuusisto J (2006). Association of metabolic syndrome with Alzheimer disease: a population-based study. Neurology.

[4] Raffaitin C, Gin H, Empana JP, Helmer C, Berr C, Tzourio C, Portet F, Dartigues JF, Alperovitch A, Barberger-Gateau P (2009). Metabolic syndrome and risk for incident Alzheimer’s disease or vascular dementia: the three-city study. Diabetes Care.

[5] Peters R (2009). The prevention of dementia. Int J Geriatr Psychiatry.

[6] Panza F, D’Introno A, Colacicco AM, Capurso C, Capurso S, Kehoe PG, Capurso A, Solfrizzi V (2004). Vascular genetic factors and human longevity. Mech Ageing Dev.

[7] Solfrizzi V, Capurso C, D’Introno A, Colacicco AM, Santamato A, Ranieri M, Fiore P, Capurso A, Panza F (2008). Lifestyle-related factors in predementia and dementia syndromes. Expert Rev Neurother.

[8] van Gelder BM, Tijhuis M, Kalmijn S, Kromhout D (2007). Fish consumption, n-3 fatty acids, and subsequent 5-y cognitive decline in elderly men: the Zutphen Elderly Study. Am J Clin Nutr.

[9] Solfrizzi V, Scafato E, Capurso C, D’Introno A, Colacicco AM, Frisardi V, Vendemiale G, Baldereschi M, Crepaldi G, Di Carlo A, Galluzzo L, Gandin C, Inzitari D, Maggi S, Capurso A, Panza F (2010). Italian longitudinal study on ageing working group. Metabolic syndrome and the risk of vascular dementia: the italian longitudinal study on ageing. J Neurol Neurosurg Psychiatry.

[10] Hashimoto M, Yamashita K, Kato S, Tamai T, Tanabe Y, Mitarai M, Matsumoto I, Ohno M (2012). Beneficial effects of daily dietary omega-3 polyunsaturated fatty acid supplementation on age-related cognitive decline in elderly Japanese with very mild dementia: a 2-year randomized, double-blind, placebo-controlled trial. J Aging Res Clin Pract.

[11] Dobbing J, Sands J (1979). Comparative aspects of the brain growth spurt. Early Hum Dev.

[12] Bazan NG, Molina MF, Gordon WC (2011). Docosahexaenoic acid signalolipidomics in nutrition: significance in aging, neuroinflammation, macular degeneration, Alzheimer’s, and other neurodegenerative diseases. Annu Rev Nutr.

[13] Gamoh S, Hashimoto M, Sugioka K, Hossain SM, Hata N, Misawa Y, Masumura S (1999). Chronic administration of docosahexaenoic acid improves reference memory-related learning ability in young rats. Neuroscience.

[14] Hashimoto M, Hossain S, Shimada T, Sugioka K, Yamasaki H, Fujii Y, Ishibashi Y, Oka J-I, Shido O (2002). Docosahexaenoic acid provides protection from impairment of learning ability in Alzheimer’s disease model rats. J Neurochem.

[15] Hashimoto M, Tanabe Y, Fujii Y, Kikuta T, Shibata H, Shido O (2005). Chronic administration of docosahexaenoic acid ameliorates the impairment of spatial cognition learning ability in amyloid beta-infused rats. J Nutr.

[16] Mills JD, Hadley K, Bailes JE (2011). Dietary supplementation with the omega-3 fatty acid docosahexaenoic acid in traumatic brain injury. Neurosurgery.

[17] Kawashima A, Harada T, Kami H, Yano T, Imada K, Mizuguchi K (2010). Effects of eicosapentaenoic acid on synaptic plasticity, fatty acid profile and phosphoinositide 3-kinase signaling in rat hippocampus and differentiated PC12 cells. J Nutr Biochem.

[18] Hashimoto M, Hossain S, Tanabe Y, Kawashima A, Harada T, Yano T, Mizuguchi K, Shido O (2009). The protective effect of dietary eicosapentaenoic acid against impairment of spatial cognition learning ability in rats infused with amyloid beta(1-40). J Nutr Biochem.

[19] Wyss JM, Fisk G, Groen TV (1992). Impaired learning and memory in mature spontaneously hypertensive rats. Brain Res.

[20] Mori S, Kato M, Fujishima M (1995). Impaired maze learning and cerebral glucose utilization in aged hypertensive rats. Hypertension.

[21] Gattu M, Pauly JR, Boss KL, Summers JB, Buccafusco JJ (1997). Cognitive impairment in spontaneously hypertensive rats: role of central nicotinic receptors I. Brain Res.

[22] Nangaku M, Izuhara Y, Usuda N, Inagi R, Shibata T, Sugiyama S, Kurokawa K, van Ypersele de Strihou C, Miyata T (2005). In a type 2 diabetic nephropathy rat model, the improvement of obesity by a low calorie diet reduces oxidative/carbonyl stress and prevents diabetic nephropathy. Nephrol Dial Transplant.

[23] Eckel RH, Grundy SM, Zimmet PZ (2005). The metabolic syndrome. Lancet.

[24] Lepage G, Roy CC (1986). Direct transesterification of all classes of lipids in a one-step reaction. J Lipid Res.

[25] Lowry OH, Rosebrough NJ, Farr AL, Randall RJ (1951). Protein measurement with the Folin phenol reagent. J Biol Chem.

[26] Ishiguro J, Tada T, Ogihara T, Murakami K, Kunihiro Y (1987). Studies on the metabolic disposition of ethyl eicosapentaenoate (EPA-E) in rats and dogs. Drug Metabol Dispos.

[27] Martins JG, Bentsen H, Puri BK (2012). EPA in major depressive disorder: eicosapentaenoic acid appears to be the key omega 3 fatty acid component associated with efficacy in major depressive disorder: a critique of Bloch and Hannestad and updated meta-analysis. Mol Psychiatry.

[28] Bloch MH, Qawasmi A (2011). Omega-3 fatty acid supplementation for the treatment of children with attention-deficit/hyperactivity disorder symptomatology: systematic review and meta-analysis. J Am Acad Child Adolesc Psychiatry.

[29] Lynch AM, Loane DJ, Minogue AM, Clarke RM, Kilroy D, Nally RE, Roche OJ, O’Connell F, Lynch MA (2007). Eicosapentaenoic acid confers neuroprotection in the amyloid-beta challenged aged hippocampus. Neurobiol Aging.

[30] Hall JC, Priestley JV, Perry VH, Michael-Titus AT (2012). Docosahexaenoic acid, but not eicosapentaenoic acid, reduces the early inflammatory response following compression spinal cord injury in the rat. J Neurochem.

[31] Chapkin RS, Wang N, Fan YY, Lupton JR, Prior IA (2008). Docosahexaenoic acid alters the size and distribution of cell surface microdomains. Biochim Biophys Acta.

[32] Hashimoto M, Hossain S, Agdul H, Shido O (2005). Docosahexaenoic acid-induced amelioration on impairment of memory learning in amyloid beta-infused rats relates to the decreases of amyloid beta and cholesterol levels in detergent-insoluble membrane fractions. Biochim Biophys Acta.

[33] Hashimoto M, Shinozuka K, Gamoh S, Tanabe Y, Hossain MS, Kwon YM, Hata N, Misawa Y, Kunitomo M, Masumura S (1999). The hypotensive effect of docosahexaenoic acid is associated with the enhanced release of ATP from the caudal artery of aged rats. J Nutr.

[34] Mori TA, Watts GF, Burke V, Hilme E, Puddey IB, Beilin LJ (2000). Differential effects of eicosapentaenoic acid and docosahexaenoic acid on vascular reactivity of the forearm microcirculation in hyperlipidemic, overweight men. Circulation.

[35] Mozaffarian D, Wu JH (2012). (n-3) fatty acids and cardiovascular health: are effects of EPA and DHA shared or complementary?. J Nutr.

[36] Ghosh A, Carnahan J, Greenberg ME (1994). Requirement for BDNF in activity-dependent survival of cortical neurons. Science.

[37] Duman RS (2002). Pathophysiology of depression: the concept of synaptic plasticity (2002). Eur Psychia.

[38] Rao JS, Ertley RN, Lee HJ, DeMar JC, Arnold JT, Rapoport SI, Bazinet RP (2007). n-3 polyunsaturated fatty acid deprivation in rats decreases frontal cortex BDNF via a p38 MAPK-dependent mechanism. Mol Psychiatry.

[39] Hashimoto K, Shimizu E, Iyo M (2004). Critical role of brain-derived neurotrophic factor in mood disorders. Brain Res Rev.

[40] Bliss TV, Collingridge GL (1993). A synaptic model of memory: long-term potentiation in the hippocampus. Nature.

[41] Moser EI, Krobert KA, Moser MB, Morris RG (1998). Impaired spatial learning after saturation of long-term potentiation. Science.

[42] Tanabe Y, Hashimoto M, Sugioka K, Maruyama M, Fujii Y, Hagiwara R, Hara T, Hossain SM, Shido O (2004). Improvement of spatial cognition with dietary docosahexaenoic acid is associated with an increase in Fos expression in rat CA1 hippocampus. Clin Exp Pharmacol Physiol.

[43] Lim SY, Suzuki H (2001). Changes in maze behavior of mice occur after sufficient accumulation of docosahexaenoic acid in brain. J Nutr.

[44] Liu S-H, Chang C-D, Chen P-H, Su J-R, Chen C–C, Chaung H-C (2012). Docosahexaenoic acid and phosphatidylserine supplementations improve antioxidant activities and cognitive functions of the developing brain on pentylenetetrazol-induced seizure model. Brain Res.

[45] McGahon BM, Martin DS, Horribon DF, Lynch MA (1999). Age-related changes in synaptic function: analysis of the effect of dietary supplementation with omega-3 fatty acids. Neuroscience.

[46] Su HM (2010). Mechanisms of n-3 fatty acid-mediated development and maintenance of learning memory performance. J Nutr Biochem.

[47] Kawakita E, Hashimoto M, Shido O (2006). Docosahexaenoic acid promotes neurogenesis in vitro and in vivo. Neuroscience.

[48] Katakura M, Hashimoto M, Hossain S, Gamoh S, Okui T, Matsuzaki K, Shido O (2009). Docosahexaenoic acid promotes neuronal differentiation by regulating basic helix-loop-helix transcription factors and cell cycle in neural stem cells. Neuroscience.

[49] Hossain MS, Hashimoto M, Gamoh S, Masumura S (1999). Antioxidative effects of docosahexaenoic acid in the cerebrum versus cerebellum and brainstem of aged hypercholesterolemic rats. J Neurochem.

[50] Green P, Yavin E (1998). Mechanisms of docosahexaenoic acid accretion in the fetal brain. J Neurosci Res.

[51] Wu A, Ying Z, Gomez-Pinilla F (2004). Dietary omega-3 fatty acids normalize BDNF levels, reduce oxidative damage, and counteract learning disability after traumatic brain injury in rats. J Neuritrauma.

[52] Hou Z, Luo W, Sun X, Hao S, Zhang Y, Xu F, Wang Z, Liu B (2012). Hydrogen-rich saline protects against oxidative damage and cognitive deficits after mild traumatic brain injury. Brain Res Bull.

[53] Wu A, Ying Z, Gomez-Pinilla F (2011). The salutary effects of DHA dietary supplementation on cognition, neuroplasticity, and membrane homeostasis after brain trauma. J Neurotrauma.

